# I don’t want to think about it: a qualitative study of children (6–18 years) with rheumatic diseases and parents’ experiences with regular needle injections at home

**DOI:** 10.1186/s12969-021-00495-4

**Published:** 2021-01-22

**Authors:** Kari Sørensen, Helge Skirbekk, Gunnvald Kvarstein, Hilde Wøien

**Affiliations:** 1 Department of Nursing Science, University of Oslo, Oslo, Norway; 2 Division of Emergencies and Critical Care, Oslo University Hospital, Oslo, Norway; 3 Department of Undergraduate Studies Lovisenberg Diaconal University College, Oslo, Norway; 4 Department of Health Management and Health Economics, University of Oslo, Oslo, Norway; 5 Department of Clinical Medicine, UiT - The Arctic University of Norway, Tromsø, Norway

**Keywords:** Needle injection, Child, Adolescent, Needle fear, Rheumatic disease, Home administration, Coping strategies, Routines, Family cooperation, Qualitative research

## Abstract

**Background:**

Overall outcomes of pediatric rheumatic diseases (RD) have improved due to treatment with biologic agents and methotrexate. For many children, this treatment often entails regular needle injections. Pain and fear of needle injections are common in childhood, but how children and parents handle long-term needle injections at home has not been fully explored. This study aimed to explore how regular needle injections affect children with RD and their parents in their daily living.

**Methods:**

This explorative qualitative study used individual interviews and focus groups to ensure a comprehensive investigation of the topic. Children aged 6 to 16 years (*n* = 7) and their parents (*n* = 8) were interviewed individually 4 to 6 months after the onset of needle injection treatment. The focus groups included children aged 11 to 17 years (*n* = 9) and parents (*n* = 8) with a minimum of 6 months of experience with injection treatment. Data were analyzed using thematic analysis.

**Results:**

The main themes; “challenges,” “motivational factors,” and “routines” captured experiences and strategies that influenced the continuation of needle injections at home. Many children feared the moment immediately before the needle stick, although they had become accustomed to the pain. Most parents felt insecure about handling needle injections and lacked follow-up from healthcare providers. The children’s experience of treatment effects and self-confidence were essential to maintain motivation for further injections. A number of coping strategies helped children focus away from injection related discomfort, often discovered by chance. Facilitating firm routines and shared responsibility within families helped children develop self-confidence during the procedure. Children and parents struggled to find suitable information on the Internet.

**Conclusions:**

Children and parents experienced long-term needle injections challenging. They used their own limited resources and cooperated within the families to create routines and to introduce coping strategies necessary to manage and keep up with the procedure. Although the injection itself was not experienced very painful, the discomfort, worries and impact on daily life represented far more than a little needle stick, and thus needs more attention from healthcare providers.

## Background

Overall outcomes of rheumatic diseases (RD) in childhood have improved substantially due to new treatment programs, including the use of biologic agents and methotrexate combined with physical activity [1–4]. Children and parents must administer most of these drugs via regular needle injections. Pain and fear of needle injections are common among children [5, 6] and may cause reluctance to use injections and non-adherence to treatment [7, 8].

Management of needle-related fear and pain has remained suboptimal even though pain management strategies are available [9, 10]. Non-pharmacological coping strategies have proven effective in reducing distress due to procedural pain and have been shown to assist children’s coping [11–15]. Even so, children are still undergoing physical restraint by parents and health care providers (HPs) when they refuse painful procedures [16, 17]. Children’s recollection of distress during procedures may cause anticipatory fear and increased pain during future procedures [9, 18]. In some cases, repeated painful procedures may lead to post-traumatic stress syndrome, non-adherence to medical treatment, and aversion to health care later in life [10, 19].

The health care of chronic illness has developed towards an increased emphasis on self-management, where a patient’s condition and the subsequent treatment are taken care of at home [20]. Children with RD and their parents normally have a short hospital stay, during which examination, initiation of treatment, and injection training take place [21]. Children and parents are expected to conduct regular needle injections at home. For children, self-management is a developmental task that starts early and changes as they grow older [22]. How children and their families handle long-term injection-based treatment may impact adherence to treatment and general self-management, and to date, this has not been fully explored. This study aimed to explore how regular needle injections affect children with RD and their parents in their daily living.

Research questions were as follows:

1. How do children and parents experience long-term needle injections administered at home?
2. What characterizes children’s and parents’ use of coping strategies at home?

## Methods

This study was part of a larger research project investigating children’s fear and pain related to long-term needle injections. The first study of the project used video observations to explore children’s pain and fear during training sessions at a hospital ward in Norway [21]. The current study used an explorative design with individual interviews and focus groups to capture the complexity of drug administration at home. Data was collected through these two qualitative methods to enlarge the width and depth of the investigation [23].

### Participants

We used a purposive sampling strategy to include information-rich cases [23]. Children aged 6 to 16 years and their parents were interviewed individually 4 to 6 months after they started using needle injections. Participants in this study had been part of the initial video observation study [21] and had consented to be invited to participate in this study. Participants from all families except one were included, and one child was represented by both the mother and father in separate interviews.

Children between 10 and 18 years with RD and a minimum of 6 months of experience with regular needle injections were included in the focus groups. The included participants varied in age, diagnosis, medication, and duration of injection experience, but sufficient group homogenousity was ensured to stimulate a climate promoting exchange of sensitive information [23]. Parents who participated in the focus groups had children under 18 years old with RD and had experience in handling needle injections at home for more than 6 months. Children and parents who participated in the focus groups were not necessarily related to each other. Recruitment efforts involved social media announcements by the Norwegian League Against Rheumatism (BURG) and the Norwegian National Advisory Unit of Rheumatic Diseases in Children and Adolescents (NAKBUR), which also provided study information to their members. Focus groups were limited to four or five children in each group and had an age span of 3 years, because the interests, experiences, and socialization of children may change substantially during childhood [24]. Parents were divided into two focus groups based on practical considerations and the fact that smaller groups work best to provide high interaction between the participants [23].

### Procedure

In-depth, semi-structured individual interviews were conducted by the first author, KS, and took place between March 2018 and March 2019. KS has long experience as a nurse working with children at different ages both clinically and in research. She formed a relation with the families during the video observation, that took place 4 to 6 months, before the individual interviews, but was not employed at the department nor involved in the regular treatment of the children. Average interview duration was 48 min (range 18–71 min) for parents and 23 min (range 14–47 min) for children. To ensure that participants felt comfortable, they were all given the option of being interviewed at home, but two children and three parents preferred to be interviewed at the hospital. The two youngest children, at the ages of 6 and 9 years, chose to have one parent present and appreciated the availability of drawing equipment during the conversation. For the remaining interviews, children and parents were interviewed separately.

Focus groups took place between March and April 2018. The author, KS, was a moderator in all groups, and HW and HS acted as secretary in two groups each. Children and parents were informed that the researchers were not involved in the treatment of the children, and that this work was associated with a doctoral dissertation. The user participant, who was 18 years old at that time, attended the two focus groups for children. She had long experience living with RD and handling injections, and could initiate some of the discussions by sharing her own story. She also took notes, which was discussed with the moderator and the secretary just after the focus groups. The average duration of focus group discussions was 70 min (range 45–100 min), and they were conducted in appropriate locations. Food and drinks were offered, and participants were engaged in ice-breaking tasks before the focus group started.

Separate interview guides suitable for adults and children of different ages were carefully developed and followed descriptions by Green & Thorogood [23], and the content were discussed in the research team and with the user participant. The main topics and questions were emailed to each family before individual interviews to initiate their preparation. The interviews were facilitated as a natural conversation, talking about the prepared topics and main questions, and the subquestions were used only if the participants did not mention the topics. The main topics in the focus groups were similar to those in the individual interviews (see Table 1). All conversations were audiotaped, and main impressions were written down immediately after each interview, while the secretary took notes during focus group discussions. Data encompassed the transcribed audiotapes and these notes. Instead of seeking *data saturation*, a concept tied to grounded theory [23, 25], we sought to include transparancy throughout the study and thorough descriptions of the sample. Malterud (2016) has proposed the concept *information power,* indicating that the more information the sample holds, relevant for the actual study, the lower amount of participants is needed [25].

**Table 1:** Content from the individual- and focus group interview guides with children

	Individual interviews with children	Focus groups with children
Introduction	Establish contact and tell about the study Talk about everyday life, something the child is interested in Offer the child to draw, write or something to puzzle during the interview	Establish contact and tell about the study Introduce each other (playing a game) Agree on some house rules: • What being said in the room is kept there, only the researchers are allowed to listen to the recordings • Don’t speak at the same time • We don’t need to agree, everyone may have different experiences Offer to write or draw
Themes and questions	Can you tell about how it is to have needle injections? • About how it feels (if it hurts, what are you doing to decrease pain or worries?) • About how it takes place (who’s doing what) Can you tell about the first time you got the injection at the hospital? • Do you remember if it was painful or if you worried? • Could anything have been done differently? Can you tell about your disease and if the injection helps you? What do children need to know when they start with needle injections? What do you think about continuing with needle injections? Is there anything else you want to tell?	Can you tell about your experiences with needle sticks? • About the frequency and length of the injection • About pain and worrying How it feels (pain and worries) If it hurts or you worry, what are your actions to decrease these? Describe what’s going on in connection with the injection • What do you do before, during and afterwards • What do the adults do? Tell about the education for needle injections In what ways do the injections affect you in school, home and leisure activities? What do you think about continuing with needle injections? What do children need to know when they start with needle injections? Is there anything else you want to tell?
Finish	Summarize the main content in the conversation and ask if I have understood it correctly Thank you very much for sharing your experiences! (Give the child a little present)	Summarize the main content in the conversation and ask if we have understood it correctly Thank you for sharing and discussing your experiences! (Give them a little present)

### Analysis

Data were analyzed using thematic analysis [26, 27], and the software tool NVivo 11 was used to structure and analyze the data. Audiotapes were transcribed by KS, and ideas for coding and analysis were noted throughout the transcription phase. Initial inductive coding of individual interviews resulted in 61 codes, which were structured into four preliminary themes and twelve sub-themes. Interviews with children and parents were coded and analyzed separately. Codes from the analysis of the interviews were used deductively to analyze the data from focus groups, while keeping an open mind to the appearance of new information. KS completed the initial coding and shared excerpts with the other authors continually. All authors met several times during the process to discuss the analysis and to redefine themes and subthemes before reaching a consensus on the final results. The analytic steps from the generation of codes to the generation of main themes have been exemplified in Table 2.

**Table 2:** Example of the pathway from codes to main theme

Codes from the individual interview data	Codes added from the focus groups data	Sub-Themes	Main theme
**Children:**	**Children:** Not Emla Relaxation Quick performance Cooling **Parents:** The child’s understanding Cooling Negotiation Physical Restraint	Coping strategies	Routines
Knowledge			
Appraisal			
Getting used to			
Distraction			
Control			
Emla			
**Parents:**			
Relaxation			
Appraisal			
Getting used to			
Distraction			
Control			
Humor			
Play			
Emla			
The child’s understanding			
**Children & parents:**	**Children:** Self-determination Having a friend present **Parents:** Support from BURG	Facilitations Daily life Prevention of side effects Shared decision making	
Regular practices			
Handling the equipment			
Teamwork			
Adjustments			
Relations			
Responsibility			
Self-injection			

*Abbreviation*: *BURG* Norwegian League Against Rheumatism

Credibility was established through broad discussions throughout the study and by including quotations from different participants in the paper [23]. Triangulation between data from different sources, including individual interviews, focus groups, and written notes validated the analysis. Member checking during interviews ensured the correct perception of participant responses, and findings were assessed by the user participant. The report of this study was guided by the consolidated criteria for reporting qualitative research (COREQ) [28].

## Results

**Fig. 1:**
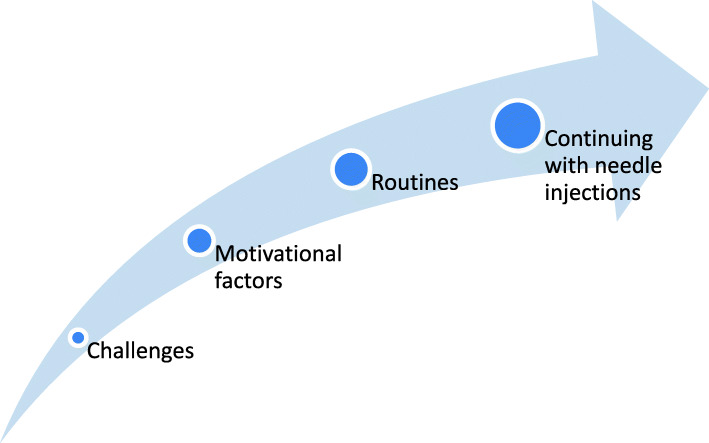
Main themes. The arrow illustrates the continuum of the three main themes capturing children’s and parents’ experiences and strategies influencing the continuation of needle injections at home. Continuation of injections at home indicates adherence to treatment

A total of 16 children (11 girls and 5 boys) and 16 parents (12 mothers and 4 fathers) shared their experiences of long-term needle injection use at home. Of these, seven children and eight parents were interviewed individually, and nine children and eight parents participated in focus groups. There was variation in rheumatic diagnosis, medication, and duration of injection-based treatment (from 4 months to 15 years). Participant characteristics have been presented in Table 3, and the source of each quotation has been marked as II (which indicated an individual interview) or FG (which indicated a focus group). Main themes have been illustrated in Fig. 1.

**Table 3:** Characteristics of study participants

Participants	Characteristics
Individual interviews	
Children:	
Gender	5 females, 2 males
Age at interview	6–16 years (mean 12 years, 4 ≤ 12 years and 3 > 12 years)
Disease duration	4–6 months (mean 5.6 months)^a^
Diagnosis	Oligo JIA (2), Poly JIA (2), Enthesitis-related JIA (1), Juvenile dermatomyositis (1), Behcets disease (1)
Medications received	Methotrexate (oral or s.c.) in combination with etanercept (Enbrel/Benepali) s.c (3) or tocilizumab (RoActemra) s.c. (1), etanercept (Enbrel) s.c. (1), methotrexate (Metex) s.c. (1) and adalimumab (Humira) s.c (1)
Parents (of the same children):	
Gender	7 females, 1 male
Focus groups	
Children (in two groups):	
Gender	6 females, 3 males
Age at time of focus group	11–13 years (mean 12 years) in the first group and 14–17 years (mean 15.8 years) in the second group.
Disease duration	6 months – 15 years (mean 8.1 years)
Diagnosis	Unspecified JIA (5), Oligo JIA (1), Poly JIA (2), Systemic JIA (1) (self-reported)
Medications received	Methotrexate (oral or s.c.) in combination with etanercept (Enbrel/Benepali) s.c (3) or tocilizumab (RoActemra) s.c. (1), methotrexate (Metex) s.c. (3), tocilizumab (RoActemra) i.v. (1) (earlier s.c. medication) and methotrexate oral (1) (earlier s.c. medication)^b^
Parents (in two groups):	
Gender	5 females, 3 males
Disease duration (child)	1–15 years (mean 7.2 years)
Diagnosis (child)	Unspecified JIA (5), Poly JIA (2), Systemic JIA (1) (self-reported)
Medications received (child)	Methotrexate (oral in combination with etanercept (Enbrel) s.c (2) or adalimumab. (Humira) s.c (1), methotrexate (Metex) s.c. (2), and methotrexate oral (2)

Number of participants: (n), *Abbreviations*: *JIA* Juvenile Idiopathic Arthritis, *s.c.* subcutaneous

^a^One child was diagnosed 10 years ago and had previous experience with s.c. injections, but after several years without s.c. injections she was readmitted 6 month earlier and needed updated education

^b^Due to severe side effects of injections or severe needle–fear converted from subcutaneous to oral administrations

### Challenges

Children and parents reported challenges associated with regular home administration of needle injections. Their stories included physical pain and emotional distress related to the injections and other painful procedures, like blood samples, joint injections, and peripheral vein cannulation (PVC). Additional challenges were connected to the handling of equipment and the prevention of side effects of the drugs.

Most parents felt insecure when they became responsible for the medical treatment of their child after discharge from the hospital, which was illustrated by the following:

“*I was thinking, ‘Oh my god – this is a huge responsibility!’ I didn’t feel competent. I have a sister who is a nurse; she gave me some advice. I thought this was unjustifiable; it should have been a nurse doing this.*” (Parent of a 14-year-old boy, II)

Many parents reported that their handling of the injections at home was hardly ever explored at regular follow-up consultations. Several parents also mentioned a lack of psychological advice on how to assist their child’s coping with pain and fear.

Children were mainly concerned about how they could avoid focusing on the needle sticks. Most children had become accustomed to the injections but reported that they still feared the moment before the needle stick even if it did not hurt much. This was even evident by children performing the injection themselves. Parents were frightened by the prospect of inserting a needle into one’s child, although a father described this as being easier than expected, “*As sliding a warm knife through butter.*” Children and parents compared the experience with other needle procedures. Most children rated blood sampling as more painful than subcutaneous injections, depending on who performed the test.

“*They are not so skilled with children at the local office. It was very painful, and I usually don’t mind blood tests at all when performed by a known person.*” (11-year-old child, FG)

Many children stated that being given sufficient information and being able to decide some parts of procedures increased their trust in those performing painful procedures. Bad experiences affected children and parents for a long time, especially if the child had been physically restrained during the procedure.

“*It almost felt like abuse, when one person held him down, another stretched out the hand and … . he still doesn’t like PVC! But he has gone a long way up until today - just need a warm hug and to squeeze my hand …* ” (Parent of 14-year-old child, FG)

Many parents reported that they admired their children for their willingness to receive needle injections, but they also talked about the difficulty of interfering with situations where HPs pressed hard to get a procedure done. One child had developed severe needle phobia after a bad experience and had refused the recommended subcutaneous treatment. Her mother felt miserable about being unable to assist her child. At the beginning of home treatment, some parents had used physical restraint, by holding their child to carry out the injections, but later realized how this traumatized both the child and themselves, and they could not continue.

Parents talked much about their efforts in handling equipment, preparing for the treatment, and implementing it. Their struggle to transfer a small drug volume from one syringe to another or to hold the correct grip sometimes resulted in spoiled drugs. Many said that they lacked written information and had to rely on their memory of oral instructions given by nurses at the hospital. Most children, however, stated that they could remember details from the training session, “*I learned that we should not stick the needle straight down, but slightly slanted*” (12-year-old child, II). Such statements indicated children’s quick learning and high self-confidence concerning technical equipment. Some parents told how their child even guided them at home.

“*She (our daughter) looks after us, that we don’t inject too slow or too fast … and told me once, ‘Mama, it’s due to the side effects we must take the injection in the evening.’ She remembers everything!*” (Parent of 12-year-old child, II)

Concerns about drug-related side effects were especially highlighted by children in focus groups. They stated that oral and subcutaneous methotrexate could cause nausea, vomiting, and tiredness. Some said that they had started feeling sick when observing something yellow, smelling or tasting anything that reminded of the drugs, or even just thinking about them. Children who were interviewed individually did not talk explicitly about such side effects, but their parents reported that precautions were taken to prevent potential discomfort. Some parents uttered their worries and mixed feelings about the drugs.

“*After all, these medicines are no good. I panic when I read about the scariest side effects. However, getting better prognosis for the disease is positive.*” (Parent of 9-year-old child, II)

### Motivational factors

The children had all suffered from pain, joint stiffness, reduced physical activity, and other discomforts to different degrees before being diagnosed. Many parents described the relief they felt when they realized that their child suffered from a treatable disease, and this motivated injection treatment. Their conclusion was that “*this is something you just have to do.*” Children’s experiences of improvement after starting treatment increased parents’ and children’s motivation to continue. Some children reported that a lack of effects or bothersome side effects decreased their motivation to continue.

“*I have taken injections at home before, but when the drug made me very sick, we had to quit (and continue with intravenous infusions of another drug).*” (13-year-old child, FG)

Another girl described how she felt psychologically tired of taking injections when she lost faith in the effect of the treatment. More examples of quotations on reported experiences of drug effects have been shown in Table 4.

**Table 4:** Children and parents’ reports of effects and side effects of the medication

Drug effects	Child’s quotation	Parent’s quotation
Positive effect	“The medication is very helpful; I’m less stiff, no pain, I’m able to do gymnastics and play handball” (12-year-old child, II)	“I’ve got a brand new girl! It happened fast, she is very happy and fresh. She felt useless (before). “(Parent of 12-year-old child, II)
Uncertain effect and side effects	“More energy, but not as much as expected. I was nauseous and dizzy” (14-year-old child, II)	“He was better in the body, could perform more, but was nauseous and had a headache” (Parent of 14-year-old child, II)
Effect and side effects	“No” (9-year-old child, II) (she had side effects of steroids)	“The results from blood samples and MR are positive. She is much stronger, she couldn’t keep her head straight” (Parent of 9-year-old child, II)
Less effect than expected	“I have had a different effect on different drugs. When having the good effect I could be with friends, join birthday parties and so on ….” (15-year-old child, II)	“It was easier when she was younger. It has been hard to find medication for her as an adolescent. She had a period where she didn’t want to take the injections – she had lost the faith in the medication.” (Parent of 15-year-old child, II)

*Abbreviations*: *II* Individual interviews

Many children reported decreased pain and joint stiffness and had more energy to attend school and perform normal sports activities. The youngest children had no clear perception of treatment effects, but their parents reported effects based on their observations. Some parents focused on the fact that their child’s probability of having a normal life was determined by a positive treatment effect, as symptoms increased during periods of adjustment or during the discontinuation of medications.

“*This is something we talk to her about – how it might have been without effective medication.*” (Parent of 13-year-old child, FG)

Parents reported how they had worked systematically to create secure environments and routines to establish a good relation and cooperation during the procedure of needle injections. Children and parents agreed that children’s self-confidence improved over time, as children knew what was going to happen. This building of positive experiences was described as an important motivational factor.

In some families, only one of the parents performed the injections, either because the other parent did not like needles, or the child did not let them do it, which was illustrated by the following: “*My mum is not allowed to give me the injection, I don’t trust her*” (11-year-old child, FG). The father in this situation reported that of the two parents, he had spent the most time building a relationship during the first injections.

### Routines and use of coping strategies

In addition to the use of routines and the building of confidence within each family, children and parents described numerous coping strategies to handle needle injections. Children and parents reported a shared experience regarding the establishment of routines and teamwork in the family. A typical example of this has been described in Table 5.

**Table 5:** A typical example of routines and teamwork described by one child and parent

Child’s quotation	Parent’s quotation
“I make everything ready and transfer 0.7 (ml) into another syringe. If there are bubbles, my mum has told me how to shake it away. I don’t dare to take the injection alone yet, mummy helps me with the needle stick and I push in the liquid. If I push too fast it’s more painful – but then I just take a break before continuing” (12-year-old child, II)	“She cried a little bit the first few times, but I was clear and told her that this is something she has to do. Little by little she has learned her routines, by first preparing the equipment, and then by sitting down and breathing for a while saying; ‘I don’t like it, but I have to do it’ – and then I insert the needle together with her” (Parent of 12-year-old child, II)

*Abbreviations*: *II* Information from an individual interview

Some children reported that having a friend, sibling, or grandparent present during the procedure decreased fear. They told how bystanders were impressed by their bravery and how they were proud of this. A total of eight children reported that they preferred to do the injections on their own and were aware of the actions necessary to become independent, such as the following participant: “*I said to myself, ‘You just have to endure this!’*” (17-year-old child, FG). Only one child, below 13 years, performed the injections herself, but two 12-year old girls claimed they would soon dare to manage the injections themselves.

They agreed that there was less pain when the injection site was in the upper part of the thigh and when the drug was tempered compared to other scenarios. Small children did not have the option to choose between different injection devices, while older children could select drug injections with either syringe or pen. Some preferred syringes, which allowed them to control injection speed, while others favored pens that completed the procedure quickly and in which the needle was invisible.

All families described how they adapted their everyday lives to minimize children’s treatment discomfort. Several children went to bed right after the injection, slept longer the following day, ate extra food, and adjusted their school and physical activities. Several children described their strategies in detail.

“*I drink tea and have a hunger for orange. I get psychological nausea and like to reward myself with some candy, listen to music, or doing something cozy.*” (17-year-old child, FG)

### Coping strategies

Most children were familiar with topical anesthesia as a pharmacological tool for pain relief, but only a few reported a significant effect. Thus, most children managed without topical anesthesia. One girl always cooled her skin with an ice cube and considered this her primary strategy. No children recalled specific instructions for non-pharmacological coping strategies. Parents used prior knowledge and their own experiences, and some searched the Internet to find methods of assisting their child. Several useful coping strategies were discovered by chance.

“*When I was going to have the injection, my favorite series was on the TV, and mom allowed me to watch.*” (9-year-old child, II)

The most commonly used coping strategies have been exemplified in Table 6. All parents offered a wide range of distraction techniques, like looking away, watching television or an iPad, talking about something else, or squeezing their parents’ hands. Most children found it helpful to focus on something else. Some reported that having the opportunity to play a video game or watch television was helpful, even when they did not use this option. Others liked to be distracted during the needle stick even if they took part in the preparation.

**Table 6:** Examples from children’s and parents’ description of commonly used coping strategies

Coping strategies	Children’s quotations	Parent’s quotations
Distraction	“I often watch TV or iPad or play a game when the injection is prepared” (Several, II)	“We have used a bunch of distraction techniques, like singing, watching movies, soft toys, cold and siblings ….” (Several, II and FG)
Rewards	“Toys, chocolate, fun adhesive plaster, poster with stickers, Lego” (Several, II and FG)	“The effect of rewards must not be underestimated” (Parent of 11-year old child, FG)
Control	“I have less control with a pen than a syringe, and I don’t appreciate that very much”. (16-year-old child, II)	“I think it has been helpful for her to decide something herself” (Parent of 16-year-old child, II)
Relaxation	“When I’m thinking of something I’m looking forward to, I get relaxed” (14-year-old child, FG)	“We practiced breathing techniques in the evenings and a bit yoga, until we felt calm and relaxed” (Parent of 6-year-old child, II)
Increasing knowledge and technical skills	“I think parents should inform their child what is going to happen, and to agree” (15-year-old child, II)	“It is easier when your child understand the reason why she needs the injection” (Parent of 8-year-old child, FG)
Pharmacological Strategies	“I used Emla before, but then I couldn’t deal with it anymore” (13-year-old child, FG)	“He doesn’t use Emla anymore – it didn’t help” (Parent of 14-year-old child, FG)

*Abbreviations*: *II* Information from an individual interview, *FG* Information from a focus group

Most children appreciated getting rewards like toys and chocolate, a nice Band-Aid with a picture on it, or a new sticker to put on a poster every time they received an injection. Children in focus groups had more experience with injections and recalled getting rewards in the beginning. Parents considered rewards a tool to negotiate with their child, and buying a hotdog in the shop at the hospital was a popular reward for completing a procedure. One father said they had used many “bribes” to persuade the child, but had to stop to be fair to the child’s siblings. Instead, the child was given the opportunity to choose an activity for the whole family, such as going to a movie, when she had received a specific number of injections.

Although negotiations and rewards were intended to provide children with control, some parents reported that this behavior delayed the procedure more than it helped the child’s coping. Gradually giving the child more responsibility was described as a better way for the child to gain control. Children wanted knowledge of the disease and needed a justification for the injections.

Some parents used metaphors, labeling the drugs the child’s best friend in helping them fight the disease. Several children had watched a video that showed a girl playing her favorite sports and living a normal life despite RD. The children found this video very helpful, and it also helped when explaining their disease to their peers. They would like to find similar videos on the Internet, which presented recommendations for the implementation of needle injections.

### Continuing with needle injections

Children accepted that they would have to continue with needle injections as long as the treatment improved their quality of life. None had received a recommendation to stop the treatment due to remission of the disease, and children and parents did not know how long the treatment would last. Some hoped to stop the treatment within a few years. Children and parents described a common goal that needle injections should become a natural part of their daily lives. They found it valuable to share their experiences of home injections, which they hoped would help other children, and stated that their experiences with needle injections were important.

## Discussion

The main findings of this study were that the children and parents encountered challenges when attempting to incorporate the injection treatment as a natural part of their daily lives. Families used their own resources and cooperated to create routines and introduce coping strategies necessary to continue with the unpleasant needle injections.

### Regular injections comprise more than a little needle stick

Short hospital stays are currently the standard for most children with chronic pediatric diseases, as the health care system focuses on self-management outside institutions [20]. However, parents in this study felt overwhelmed by their responsibility for the daily treatment, which included technical and emotional challenges related to the injections. Findings indicated that many families may need additional follow-up sessions and a gradual increase in responsibility before they are capable of taking care of their child’s needs during the needle injection procedure at home.

The parents’ views on the treatment varied from being optimistic about treatment effectiveness to worries about potential side effects and long-term consequences. Gomez-Ramirez and colleagues found similar mixed positive and negative emotions among parents of children with juvenile idiopathic arthritis (JIA), who they described as being on a rollercoaster ride [29]. Children in the present study rarely described the same emotional ups and downs as those reported by young people with juvenile dermatomyositis [30]. In this study, children may have answered questions about the circumstances of needle injections in a narrow sense, while parents shared their stories about the total situation related to having sick children.

Most children and parents gained increased confidence regarding the needle injections over time. However, home treatment entails risks and challenges, such as parents restraining their child or drugs being handled incorrectly, which may disturb treatment continuation. Our findings indicated that home-administration of needle injections is a vulnerable situation that may require individual follow-up by HPs. This was in line with the findings of two previous qualitative studies [29, 30].

Most children feared pain from the needle sticks, although they reported that it did not hurt much. Pain and fear due to needle sticks are common among children [5] and may result in fear, negative pain memories, and needle phobia lasting into adulthood if poorly managed [9, 31]. Although children who were interviewed seemed to adapt to injections over time, many reported fear just prior to the stick, and a few parents reported that severe needle phobia obstructed the treatment. Needle fear may develop after frightening or painful experiences and is linked to operant and respondent learning processes alongside changed transmission and modulation in the nervous system [9, 18]. Researchers have emphasized that a child’s memory of pain from the first needle injection may be more important for future experiences of pain and distress than the pain itself [9, 18]. In this study, some children who participated in the video observation study showed more fear during the first injection [21] than they recalled in the interviews, suggesting that later on, they may have reframed their memories in a positive direction.

Reframing children’s pain memories immediately after a procedure may reduce anticipatory fear and can be facilitated by telling children how brave they were and how they have done a good job for their body or by boosting their self-efficacy in terms of coping [31]. Many parents in this study had worked hard to build positive experiences for their child related to the injection procedure. Children’s confidence at the time of the interview was developed by initial actions at the hospital and parents’ support at home. Children and parents emphasized how routines and mutual trust were essential to build confidence and decrease distress. Routines allowed children to become familiar with equipment and the injection. This approach resembled an exposure-based exercise, as it involved allowing children to play with the equipment, which has been shown to reduce high levels of needle fear [32, 33]. Findings revealed that some children found it easier to perform the procedure with bystanders present, but others had to be alone or trusted only one parent to perform the injection. This suggests a necessity to individualize routines.

### Distraction may not be the preferred coping strategy

Children and parents gave detailed information about their coping strategies for handling needle fear. Researchers have recommended distraction as the preferred coping strategy for children during needle procedures [12, 15, 34–36]. In the present study, distraction was found useful in the beginning, especially among the youngest children, as it helped children to focus away from the needle stick. Parents often stated that the effect of distraction was discovered by chance and tried out intuitively rather than in response to explicit advice from HPs. Most research on distraction has been related to needle procedures delivered by HPs in time-limited contexts, such as vaccination clinics or during PVC procedures, which are different from home settings for long-term needle injections treatment. Distraction is easily applied in various contexts, and there is a variety of distraction methods available, including iPads, singing, televisions, looking away, talking to other people, squeezing someone’s hand, and the application of something cold. Music, bubbles, medical clowns, virtual reality, sweet-tasting solutions for infants, and devices that produce cold sensations and vibrations (for example, “Buzzy”) have been shown to be effective in previous research [13, 37–39]. Computer tablets (iPads, iPods, and smartphones) are popular among children and easy to use.

However, a recent randomized controlled trial of distraction using computer tablets for 41 children aged 4 to 11 years who underwent immunization, found that increased pain and negative emotions were reported in the intervention group [40]. Previous studies have suggested that the effect of distraction is not only explained by the method itself but also by the child’s perceptions of control [41, 42]. Children with cystic fibrosis and their parents, reported that taking control was essential in coping with needle procedures, meaning the child had to decide some parts of the procedure [42]. Nurses stated in another qualitative study that the child’s feeling of control was the basis for successful use of distraction [41]. In our study, some children stated explicit that they needed to feel in control and did not like distraction, whilst others described how they combined control and distraction. The findings of the present study support previous research, highlighting children’s perceptions of control as an important part of non-pharmacological methods. Giving children the opportunity to participate in preparation and implementation of the needle procedure seems to be especially meaningful for children with chronic diseases, who are subjected to many painful procedures.

The provision of adult support through non-procedural talk and humor as a means of distraction, has been shown to be effective in improving children’s coping during painful procedures, whilst reassuring comments, criticism, apologies, and entrusting children with too much control may increase distress [43]. Parental coaching requires training of the parents, and children with high levels of fear may also need professional support [44]. Research has shown that parents are often given information and supervision immediately before a procedure takes place, when they are distressed [45]. This may limit a parent’s memory of the training, meaning that they may need additional training to feel confident, especially when acting as a coach for their child. In general, parents of children with chronic diseases are at risk of acting too protectively, which may decrease their child’s self-efficacy and augment somatic symptoms [46].

Coping strategies must be age-appropriate. For instance, sweet-tasting solutions are highly recommended for infants, but these are found ineffective in school-aged children [13]. Offering sweets during or after injections was relatively common in this study, and children appreciated sweets and other rewards. Many parents expressed critical comments on this practice, because rewards were unfair to siblings and probably delayed the procedure rather than assisting their child’s coping. Children, however, reported positive experiences of rewarding themselves by thinking about something pleasant or doing something fun or cozy. Rewards may be an easily available and commonly used coping strategy unless children and parents receive education on other strategies. Research on the use of rewards is sparse compared to research on distraction techniques, but one study found that parents used rewards after almost 90% of immunizations, whilst distraction was offered during 15% [47]. Findings from the present study showed that rewards may not be the best coping strategy in the long-term.

### Building confidence in everyday life

Our findings supported the findings of studies that emphasized children’s need to participate in health care decision making [35] in settings where needle procedures are repeated over a long period of time. Although most children seemed to adapt to active coping strategies over time, many families strived until they found a suitable and stable strategy. Some parents who participated in focus groups had used physical restraint in the beginning, because they lacked appropriate coping strategies. This confirmed that physical restraint for painful procedures is still in use. HPs continue to believe that getting a procedure done quickly is preferable for the child despite growing evidence of harmful effects, especially when conducted by parents [17, 19]. Parents are often given the role as “helper” for HPs instead of being prepared for the role of comforting and supportive of the child [16].

Holding a child physically during medical procedures may increase pain and distress during the current procedure and in future procedures and is strongly advised against [48]. The fact that physical restraint was not reported in individual interviews may indicate a change in clinical practice and better awareness of this topic. Focusing on adult communication, acknowledging children’s fear, and supporting engagement may strengthen the choice of coping strategy and improve children’s decisional control [21]. Many families experienced a very brief education during short hospital stays, which gave them insufficient confidence to handle the technical and emotional challenges associated with the injections. They described a need for far more support and follow-up, and they lacked appropriate information about available material on the Internet.

The importance of participation in school and physical education among children with JIA was shown in a recently published longitudinal study [49]. In this study, school absence at the onset of the disease predicted poorer quality of life several years later. Our findings revealed that children made much effort to maintain school attendance. In general, they used weekends for drug administration to diminish the burden of potential side effects, although this strategy might impede social activities. Methotrexate intolerance was highlighted by children in focus groups, and many parents explained how they took precautions to prevent undesired drug effects.

Methotrexate intolerance may be particularly evident among children with JIA [50], and there seems to be a strong positive association between side effects of methotrexate and needle pain [51, 52]. This intolerance was not the main topic in this study, but findings indicated that intolerance played an important role in children’s experiences with needle injections. In an interview study of 12 children (aged 6–12 years), the authors concluded that methotrexate treatment was more difficult than other painful procedures and highlighted the importance of strategies and routines to manage medical side effects [53]. In contrast, in the present study, blood tests were reported more painful than subcutaneous injections. One explanation for this distinction may be the firm routines families had established at home. Children did not have to worry about variations in everyday injection procedures, while blood tests could be performed in unpredictable ways.

### Strength and limitations

This study had some limitations. First, individual interviews were conducted a relatively short time after injection treatment had begun, and challenges and coping strategies might have been different if participants had a longer experience or if they had been interviewed a second time. However, focus groups provided a robust longitudinal perspective of children’s and parents’ experiences. Second, families who volunteered for focus groups were generally resourceful with only moderate problems. However, several parents talked freely about their child’s fear of needles and the challenges they faced, such as using physical restraint during injection treatment. A strength of the study was that children of different ages and parents were allowed to share their experiences. Finally, gender might have influenced the results, but using this chosen qualitative approach studying gender differences is not suitable.

## Conclusion

Children and parents strived to make the home administration of needle injections a natural part of daily living. Parents felt thrown into a huge responsibility and did their best to preserve their child’s trust and cooperation during injections. Most of the learning process and the development of self-management took place at home. Fear of needle pain was present among children, even though they reported that the injection caused only slight pain. Children’s main wish was to think as little as possible about injections and to participate in normal activities, as healthy children do. Individual facilitation and choice of coping strategy, the creation of firm routines, and taking shared responsibility in families seemed to improve confidence with long-term injections and seemed to be as important as coping strategies themselves. However, confidence depends on several factors and changes over time, as the child grows older. Regular interest, as well as focus and assessment from HPs on how needle injections are handled at home, would probably serve children’s and parents’ confidence and overall self-management over time.

This study confirmed findings from a previous study [21] that emphasized the importance of the quality of the first training session and the need for follow-up sessions related to needle injections after being discharged from the hospital. The first training session and follow-up session are often performed by nurses alongside a physician consultation. Investigations of nurses’ qualifications and organizational preconditions to conduct education and follow-up sessions on needle injections are needed. This study also illustrated the complexity of regular needle injection treatment at home and its difference from painful procedures completed in the health care services. Future research should focus on interventions that support children’s and parent’s resources and individual needs at home.

## Data Availability

The dataset (audiotapes, transcriptions, and notes) have been stored at TSD at the University of Oslo and have not been made publicly available. This is due to the high risk of identification of the participants.

## Abbreviations

RD: Rheumatic diseases
JIA: Juvenile idiopathic arthritis
II: Individual interview
FG: Focus group
HP: Health care provider
